# Alginate- and Chitosan-Modified Gelatin Hydrogel Microbeads for Delivery of *E. coli* Phages

**DOI:** 10.3390/gels10040244

**Published:** 2024-04-02

**Authors:** Farzaneh Moghtader, Sencer Solakoglu, Erhan Piskin

**Affiliations:** 1 NanoBMT: Nanobiyomedtek Biyomedikal ve Biyoteknoloji San.Tic., Ltd. Sti., 48800 Köycegiz, Mugla, Turkey; erhan.piskin@nanobiyomedtek.com.tr; 2 Feyzciftligi A.S., 16700 Karacabey, Bursa, Turkey; sencers@feyzciftligi.com; 3 TiPHAGE San.Tic. A.S., Teknopark İstanbul, 34906 İstanbul, Marmara, Turkey

**Keywords:** gelatin hydrogel microbeads, modifications, sodium alginate, chitosan coating, *Escherichia coli*, T4 phages, phage loading, release and storage stability

## Abstract

Bacterial infections are among the most significant health problems/concerns worldwide. A very critical concern is the rapidly increasing number of antibiotic-resistant bacteria, which requires much more effective countermeasures. As nature’s antibacterial entities, bacteriophages shortly (“phages”) are very important alternatives to antibiotics, having many superior features compared with antibiotics. The development of phage-carrying controlled-release formulations is still challenging due to the need to protect their activities in preparation, storage, and use, as well as the need to create more user-friendly forms by considering their application area/site/conditions. Here, we prepared gelatin hydrogel microbeads by a two-step process. Sodium alginate was included for modification within the initial recipes, and these composite microbeads were further coated with chitosan. Their swelling ratio, average diameters, and Zeta potentials were determined, and degradations in HCl were demonstrated. The target bacteria *Escherichia coli* (*E.coli*) and its specific phage (T4) were obtained from bacterial culture collections and propagated. Phages were loaded within the microbeads with a simple method. The phage release characteristics were investigated comparatively and were demonstrated here. High release rates were observed from the gelatin microbeads. It was possible to reduce the phage release rate using sodium alginate in the recipe and chitosan coating. Using these gelatin-based microbeads as phage carrier matrices—especially in lyophilized forms—significantly improved the phage stability even at room temperature. It was concluded that phage release from gelatin hydrogel microbeads could be further controlled by alginate and chitosan modifications and that user-friendly lyophilized phage formulations with a much longer shelf life could be produced.

## 1. Introduction

Bacterial infections are among the most significant health problems/concerns worldwide. Antibiotics changed medical practice by significantly decreasing the morbidity and mortality associated with bacterial infections. In general, antibiotics have been successfully applied to fight against pathogens for a long time; however, now we are faced with limitations due to the emergence of antibiotic-resistant bacteria, which is a public health challenge with extensive health, economic, and societal implications [1,2,3,4]. WHO stated that “Antibiotic resistance is one of the biggest threats to global health, food security, and development today—antibiotic resistance can affect anyone, of any age, in any country”.

Phages are nature’s antibacterial agents—the most abundant living organisms in the world, being typical viruses. They quite specifically infect, replicate, and kill/ destroy their target bacteria; however, they are known as harmless to humans. They demonstrate several advantages over traditional antibiotics, including high specificity, self-replication, and a low likelihood of inducing bacterial resistance [5,6,7,8,9,10,11,12,13,14]. Therefore, they have great potential as antibacterial agents in diverse applications, such as medical therapy and cosmetics; agriculture, for the treatment of infected animals and plants; and food and environmental safety, as important alternatives to antibiotics [15,16,17].

Phages are composed of several proteins with different structural/functional properties in unique 3D structures—they are prone to denaturation and may be adversely affected/inactivated at the sites where they are prepared, stored, and used. Maintaining their activity and effective usage is an important concern. Controlled release—both the release rate and mode—are important requisitions/expectations in many applications and should be carefully considered and optimized in a tailor-made fashion depending on the target uses. Phages have been entrapped within carriers, mostly biobased/biodegradable polymers, mainly in spherical forms. Here, we have attempted to prepare polymeric microspheres using natural polymers—mainly gelatin (GEL), sodium alginate (SA), and chitosan (CS) in different formats.

Gelatin is one of the most successful and widely studied natural polymers. A protein/polypeptide—a denatured form of collagen—has been prepared in various forms for diverse applications. Gelatin cross-linked hydrogel beads have been successfully designed/fabricated for controlled- or sustained-release matrices, especially for environmentally sensitive molecules, such as several growth factors for various medical applications [18,19,20]. There are several advantages to using gelatin matrices in these kinds of biomedical applications. Gelatin is a hydrolyzed form of native collagen: it is highly biocompatible and has the property of being degraded and easily absorbed in the body, and it is commercially available at low costs. Gelatin matrices could be easily shaped and then cross-linked at different levels (means) with different cross-linking densities to form hydrogels, which swell in water quite rapidly and suck up the aqueous surrounding phase with its content, meaning that they could be easily loaded with very high loading efficiencies (almost 100%) of water-soluble substances at very mild conditions without inactivation of biological molecules such as growth factors.

**Figure 1:**
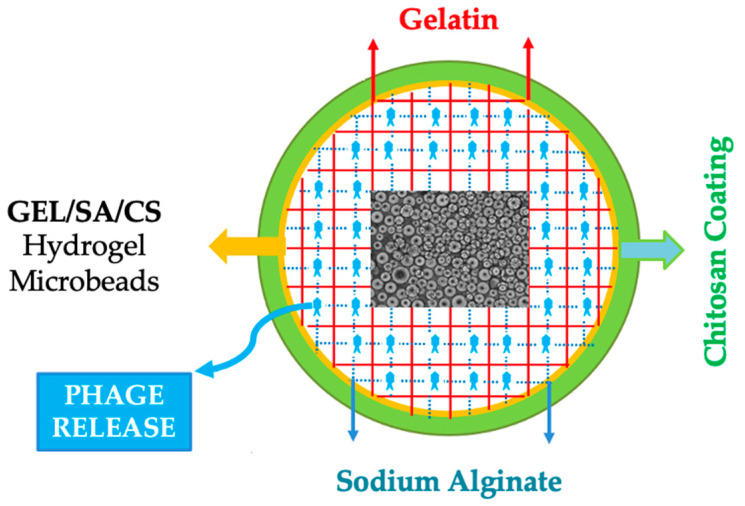
The schematical drawing of the GEL/SA/CS hydrogel microbeads prepared in this study for phage delivery.

In our related recent studies, we have prepared gelatin hydrogel microbeads—cross-linked using several cross-linking methods, including de-hydrothermal treatment (by heating under vacuum), which is the method that was also utilized in the present study [21,22,23,24]. We have demonstrated that high phage release rates are achieved as predicted for the successful prevention of open wound infections. Here we have attempted to reduce the phage release rates for the conditions requiring longer therapies such as chronic wounds with severe infections. The gelatin microbeads were modified with alginate and chitosan, both of which are natural polysaccharides, polyanionic and polycationic, respectively. They are well-known natural polymers and have been widely used in diverse bio-applications [25,26,27,28,29]. Figure 1 presents the schematical drawing of the GEL/SA/CS hydrogel microbeads attempted to be prepared in this study for phage delivery.

We have demonstrated that phages could be loaded effectively in alginate macro-beads (a few mm in diameter) and chitosan can be used for encapsulation of those beads for further stabilization and sustained release in the gastrointestinal tract [29]. Here, we prepared microbeads (around 40–50 microns in diameter), which were cross-linked physically (by de-hydrothermal treatment). Either pure gelatin or gelatin + sodium alginate mixtures were utilized to obtain the respective gelatin or gelatin + alginate hydrogel microbeads. Phages were loaded within both of these beads and the gelatin + alginate microbeads were further encapsulated with chitosan by polyelectrolyte formation (between surface alginate groups (negatively charged) and chitosan (positively charged) after phage loading. Preparation and phage release modes are demonstrated comparatively as presented below.

Phages may lose their activity in preparation, storage, and/or use due to environmental stresses, i.e., changes in temperature, pH, ionic strength, mechanical stresses (like mixing and agitation), and exposure to denaturants (like organic solvents) [30]. Several approaches have been proposed to prepare phage formulations with long shelf lives and efficient and practical forms, which include preparation of dispersions in proper growth media (like Luria-Bertani (LB) broth, trypticase soy agar, and brain heart infusion broth) or buffers (e.g., SM and phosphate buffer saline-PBS) supported with several active and stabilizing ingredients (amino acids, sugars, etc.) and microencapsulation within several natural and synthetic polymeric materials (like liposomes, alginate, chitosan, and polyhydroxy acids); spray and freeze-drying [31,32,33,34,35].

Lyophilization (freeze-drying) is routinely utilized in the pharmaceutical industry for the production of several dry products, including several proteins and vaccines. It is also the most popular technique for preparing phage formulations, which is the basic methodology that we have applied in this study [35,36,37,38,39]. As reported, phages may lose their activity during lyophilization, mainly depending on process conditions. Especially, the freeze-drying step is critical—during freezing of water in the medium and in other terms, forming ice crystals causes significant changes and increases in viscosity and osmolality, which adversely affect phage morphology, health, and activity. Therefore, a series of substances have been studied as stabilizers/cryopreserves, including soluble gelatin, and included in the recipes before lyophilization [36,37,38]. Here, instead of using gelatin in solution, we have loaded T4 phage within the gelatin hydrogel microbeads during swelling as an alternative to excipient use.

## 2. Results and Discussion

### 2.1. The Target Bacteria (E. coli) and Its T4 Phages Propagated

*Escherichia coli* (*E. coli*) was used both as the host for the propagation of the T4 phages and as the target bacteria for the phage activity and performance tests. The *E. coli* strain used here was obtained from the American Type Culture Collection (ATCC^®^ -Manassa, VA, USA) and cultured within an LB medium. In the bacterial culture medium, there was a time lag of about 1 h, and then steady-state growth was achieved. The optical density (OD_600_) of 0.5 corresponds to roughly a bacterial concentration of 10^8^ CFU (“Colony Forming Unit”)/mL, which was very efficient for propagating T4 phage emulsion with high concentrations for further studies.

**Figure 2:**
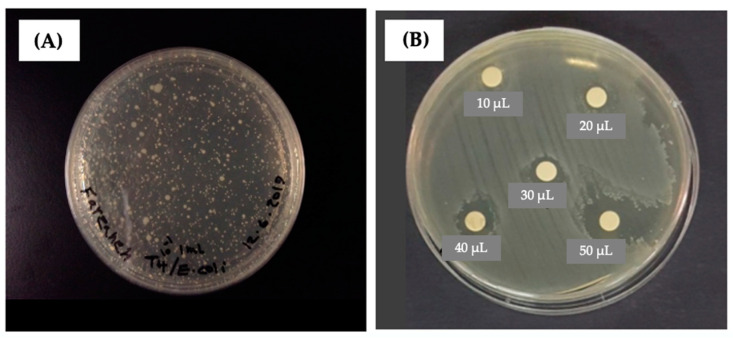
(**A**) A typical plaque assay test result to obtain the phage titers; (**B**) a typical agar over-layer test result that demonstrates the activities of T4 phages propagated in this study. The diameter of the clear zone increased with the volume of phage emulsion (with the same phage titers) dropped on the bacterial layer in the agar medium [23].

T4 phage is one of the seven phages that infect *E. coli* with high specificities; it was also obtained from ATCC^®^ (Manassa, VA, USA) and propagated using *E. coli* as the host in an LB medium. It was possible to reach very high phage concentrations (titers) up to 10^9^–10^10^ PFU (“Plaque Forming Unit”)/mL with the protocol applied here. The image in Figure 2A demonstrates a “plaque assay” result performed to obtain the phage concentration after propagation.

Figure 2B gives a representative “soft-agar overlayer assay” and the phage activity test result. The turbid areas are the bacteria grown on the soft-agar layer while the transparent zones/circles show the areas where the phage emulsions were dropped. It should be noted that the diameter of the clear zone is related to both the amount of phages dropped in that zone and their activities. It was observed that the diameter of the clear zone increased with the volume of phage emulsion (with the same titers) dropped on the bacterial layer on the agar medium.

**Figure 3:**
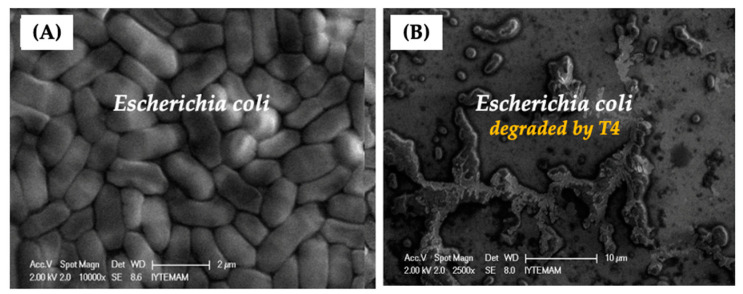
(**A**) The representative SEM images of *E. coli* (the inset image); (**B**) the destruction of *E. coli* by T4 phages dropped on the bacteria on the surface [23].

The scanning electron microscope (SEM) images of *E. coli* provided in Figure 3A show that they are homogeneous in size and cylindrical in shape and retained their original form during SEM imaging. After we dropped the T4 phages on the top of the *E.coli* layers on the SEM substrate surface, they quickly started to destroy their target bacteria, and the process was completed in about 20–30 min—the entire surface acquired an oily appearance, with almost no intact bacteria left as demonstrated in Figure 3B.

### 2.2. Gelatin-Based Hydrogel Microbeads Produced

**Figure 4:**
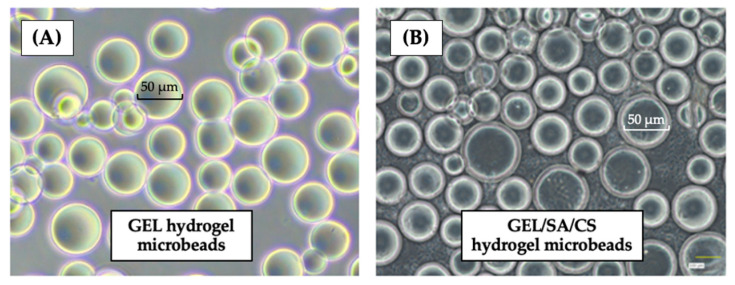
The representative optical images: (**A**) the GEL hydrogel microbeads; and (**B**) the GEL/SA/CS hydrogel microbeads [23].

The gelatin (GEL) and the gelatin/sodium alginate (GEL/SA) microbeads were prepared by a two-step process with the recipe and processing conditions optimized in our earlier studies following the related studies of our previous collaborator, Tabata’s group, to produce hydrogel microbeads [18,20,21,23]. There was a size distribution as expected, which was due to mechanical stirring applied in the gelation reactor at the first step; therefore, we fractionated the outcome as mentioned before to obtain beads with narrower size distributions. The second step involved a de-hydrothermally cross-linking protocol for 24 h, as previously reported in the related literature. Sodium alginate was included in the initial recipe to produce the GEL/SA hydrogel microbeads, and then the same de-hydrothermal cross-linking was applied for only 24 h—this is the first presentation of these composite microbeads with this protocol in the related literature. Note that chitosan coating was performed after phage loading in the finishing step. Representative optical images of these gelatin-based hydrogel microbeads are shown in Figure 4. Note that we took several pictures of the swollen beads prepared in the same or different batches and from different parts—they were all different because of the size distribution in all productions—therefore, these images here are only representative. Notice that hydrogel beads were spherical in shape and had smooth surfaces but retained size distribution even after fractionation as expected.

Swelling (water uptake) of the gelatin-based hydrogel microbeads was obtained and presented here as the water content (by weight percentage). Table 1 provides these values plus the average diameters of the beads obtained after swelling in water. As seen here, the swelling ratio of the GEL microbeads is almost 99%. The average diameters of these swollen microbeads were around 50 µm, which was in the targeted size range for this specific study, which was the result of the recipe and processing conditions that we applied. While the water uptake of the SA-modified gelatin-based hydrogel microbeads (i.e., the GE/SA microbeads) was lower—about 90% and 85% for these composite beads prepared with the GEL/SA ratios of 1.1/0.1 and 1.0/0.3 *w*/*w*, respectively—increasing SA relative amount in the initial gel mixture resulted in tighter/less swellable cross-linked networks and the average diameters of those microbeads around 40 µm. It should be noted that there were almost no changes in the GEL/SA microbeads after chitosan treatment.

It should be noted that gelatin is a polypeptide and sodium alginate is a polysaccharide—both form hydrogels but have different backbone structures. It is not easy to describe the fate of cross-linking. 

There are already several functional groups on both polymers, which means that they could react with each other at different sites on their backbone chains and form the network structures. The de-hydrothermal treatment is a physical cross-linking approach applied in a vacuum at 140 °C. It should be noted that two competing processes may occur during this de-hydrothermal treatment: (i) degradation of the gelatin backbone chain (break-offs) and (ii) cross-linking (bond formation between the chains, inter and/or intra). Therefore, not only the cross-linking degree but also the network/cross-linked structure of the final matrix may change significantly as a result of those two opposite effects, meaning that the change both in the water content and in the swollen diameter would be different as we see in Table 1. These data show that including SA in the GEL recipes for the protocol that we have applied here results in less swellable and smaller hydrogel microbeads. Therefore, the release rate of the active agents (here T4 phages) was lower (slower), which was the main foresight of this study; adding SA in the GEL hydrogel microbeads to decrease the release of phages is further discussed in the following section.

The Zeta potential of the microbeads is also provided in Table 1. We used basic gelatin (known also as Type A) with an isoelectric point (IEP) of 9.0, which means that it is positively charged at the physiological pH (7.4). However, when we introduced SA into gelatin in the preparation of the GEL/SA, the surface positive charges were reduced, mainly due to carboxylic acid groups coming from alginate. This effect was more pronounced when the SA content increased—even Zeta potential was negative. The surface charges of the GEL/SA/CS microbeads were positive as expected, due to the incorporation of positively charged chitosan molecules that interacted with the surface alginate molecules and created extra charge on the bead surfaces. These changes in Zeta potentials could be considered indications of the incorporation of both SA and CS molecules within the GEL microbeads. However, it should be carefully noted that gelatin, alginate, and chitosan are complex natural macromolecules; functional groups like amino, amide, carboxylic acid, etc. change with the raw material source/processing conditions, their molecular weights, degree of deacetylation in chitosan, preparation of microbeads, the type of cross-linking protocol/conditions, pH, ionic strength, and existing ions with different charges within the medium that they are used in. This is a quite complicated and interesting case, not intended to be discussed in this article [40,41,42,43,44,45,46,47,48,49,50,51,52,53].

### 2.3. Acidic Degradation of Gelatin-Based Hydrogel Microbeads

**Figure 5:**
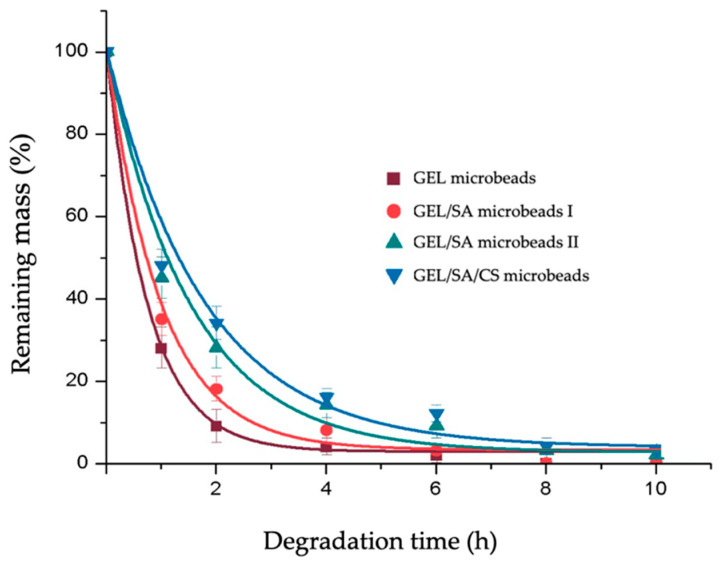
Acidic degradation of the gelatin-based hydrogel microbeads prepared in this study.

Here, acidic degradations of the gelatin-based microbeads with different physical and chemical network structures were investigated in HCl to compare their responses to an acidic environment that could be different depending on the cross-linking methodology applied, cross-linking degrees, and network structures as reported in the related literature [21]. Figure 5 summarizes acidic degradation data. As seen here, the degradation of the GEL microbeads in HCl was fast, and almost 90% of the matrices were degraded in 2 h. The addition of SA in the initial recipe created observable differences: degradations of the GEL/SA microbeads were slower and were more noticeable when the content of SA increased. The chitosan coating decreased the degradation further but not that significantly. The differences in Figure 5 could be only the differences in the network structures and possibly a small buffering effect of positively charged chitosan, which was not that significant here because it was only a partly coated layer, which was not pure chitosan but a polyelectrolyte complex.

Despite the differences in the initial period of degradation, all the gelatin beads with different network structures were completely degraded in this highly acidic medium in about 6–10 h. It should be noted that the microbeads were in the dried form when we started these tests; they were allowed to swell in the degradation medium (adsorbed the acidic medium) and started to degrade. We designed this degradation test to eliminate the diffusion limitation of acid into the matrices and aimed to see only the differences in acid–matrix interactions. In other terms, the degradation data reflect only the acidic degradation of the gelatin and/or alginate backbones without diffusion limitation.

Acidic degradation of gelatin microspheres that were produced by very similar recipes and processing conditions, cross-linked by physical (like de-hydrothermal treatment) or chemical (for instance using mainly glutaraldehyde as the cross-linking agent) agents, have been already reported, comparing the effects of cross-linking methods and degrees. It should be noted that the degradation behavior/rate of the GEL microbeads that we produced in this study was very similar to the results reported in those studies [21]. However, other matrices (not only GEL microbeads) that we prepared here were quite different; we included sodium alginate in the recipes, with chitosan to coat the microbeads in the final finishing step.

The solution extrusion of sodium alginate within the dispersion medium containing divalent cations like Ca^+2^ is a very simple approach to producing alginate hydrogel beads with uniform spherical forms, which could be achieved at room temperature [25,26,29]. Alginate beads are pH-responsive: they exhibit a gel phase transition at around pH 2.5–3.0 and they shrink (collapse) at values lower than this pH, such as the pH of the gastric juice, which is 1.5–2.0 in humans. However, the intraluminal pH rapidly changes from highly acidic in the stomach to about 6.0 and then gradually to 7.4 in the intestine. Alginate beads are expanded in the intestine and release their cargo rapidly. In conclusion, alginate hydrogels were studied for oral administration of different, especially pH-sensitive drugs, including phages for the GI sustained-release pharmaceutical formulations [25,26,29,42,43,44]. In many of these studies, alginate hydrogel beads were further stabilized by successful coating with natural (chitosan) and synthetic (polyethylene imine) polycations [27,28,29,42,43,44,45]. The data that we present are different—we have microbeads (around 40–50 μm) and the main matrix is gelatin, but the previous ones were beads with a few mm in diameter made of alginate but with polyelectrolyte coats (including chitosan) for further stabilization in acidic pH [29].

Alginate hydrogels have been widely used in biomedical applications. One of the limitations is their very slow in vivo degradation compared with gelatin, because of the obvious fact that alginate is a polysaccharide and does not exist in human tissue—our bodies do not know how to handle it. While gelatin is a denatured form of collagen, which is the main protein (polyaminoacid) in the human extracellular matrix, there are enzymes (that could be synthesized) that degrade collagen and, also, gelatin in our biochemical life cycle through extremely well-described pathways and timing. It is not possible to extrapolate the acidic degradation curves that are presented in Figure 5 and propose an in vivo degradation behavior of our gelatin-based microbeads in tissues. Any further comments will be overestimation/not very realistic.

In some applications, like the gastrointestinal sustained-release of phage formulations, acidic degradation is considered an important issue as discussed above. However, another important note should be pointed out. There are a number of studies reported to lower the molecular weight of alginate—in other terms, to make the backbone of alginate degradable by acid hydrolysis, enzymatic degradation, gamma irradiation, and some oxidation technology [45,46,47,48,49,50,51,52,53]. In this study, we apply de-hydrothermal cross-linking, which causes degradation of both gelatin and alginate backbones; however, they bind to each other by intra- or inter-cross-linking bounds and form a non-soluble network structure. The degradation data presented above are in HCl at room temperature, as in vitro degradation test; however, when the matrix starts to degrade in vivo, the gelatin chains will be degraded—most probably by enzymatic cleavages—and the released alginate molecules will be much smaller than alginate used at the beginning in the hydrogel microbead preparation. This is an important clue and will be considered carefully in our animal model degradation studies, which are under investigation.

**Figure 6:**
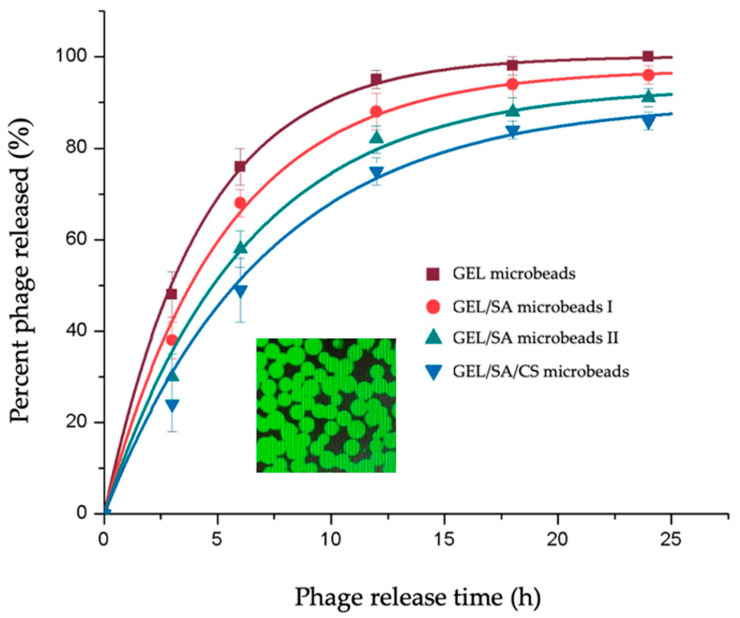
Phage release from the gelatin-based hydrogel microbeads prepared in this study. The FITC-labeled T4 phages loaded within the GEL microbeads are seen in the inlet for demonstration [23].

The T4 phages propagated in the previous steps were loaded within the gelatin-based hydrogel microbeads by following a very simple protocol. The dry beads were soaked within the emulsions of the phages, and while they were swelling, they absorbed all the aqueous phase around containing the phages, meaning that the loading yield was almost 100%, which is usually an important issue/limitation in loading of active agents within carriers. The inlet image in Figure 6, a representative picture of the GEL hydrogel microbeads carrying the FITC labeled phages, demonstrates homogeneous/effective loading.

The release from the GEL microbeads was fast—almost 80% of the phages loaded were released in 6 h, and the release was completed in 24 h (Figure 6). It should be noted that most open wounds are prone to bacterial attacks in the hospital environment; therefore, speedy actions are needed to prevent infections and initiate phage activity events as early as possible. This means that the GEL microspheres carrying phages are suitable formulations to reach this target. However, in some cases, like tissue engineering biohybrids for combined therapies of severely infected wounds with large tissue losses, one may desire lower release rates for a sustained release of the loaded phages. The original objective of this study was to decrease the phage release by the addition of sodium alginate in the microbead production recipes and chitosan coating.

As seen in Figure 6, including SA in the recipes has a significant effect. Increasing the relative amount of alginate from the GEL/SA ratio in the initial recipe from 1.0/0.1 to 1.0/0.3 *w*/*w* considerably decreased the phage release rate. As already presented in Table 1 above, the water uptake by the GEL/SA microbeads is lower than that by the GEL microbeads, and their diameters are smaller, which means that the cross-linking density and network structure of GEL/SA microbeads are different and tighter. The diffusion rates in these tighter network structures could be the result of the diffusion resistance in these matrices, which leads to lower release rates.

We also attempted to use chitosan to coat the GEL/SA beads in the final finishing step in bead preparation in order to further decrease the phage release rates. In this case, it seems that a kind of plateau was reached—almost 15% of the phage remained in the matrix even after 24 h. We originally expected much slower release rates due to the positively charged chitosan coating. However, it seems that the effect is not that significant in our experimental conditions here. Chitosan-alginate layers are formed as a result of polycationic and polyanionic chains between CS and SA chains, respectively—mainly to form much more stable encapsulation walls—as we have achieved in our related studies to reach the whole compact coating layer on the alginate beads [23,29]. As we depicted schematically in the Graphical Abstract, the situation here was different, with the CS coating forming an additional barrier to phage diffusion out of the beads. However, this did not completely prevent the release, which may be the result of having a patchy coating instead of the whole coating layer of chitosan on the beads, as demonstrated in the Graphical Abstract.

The sustained release observed with the GEL/SA and GEL/SA/CS hydrogel microbeads could also be an indication of phage–matrix interactions, most probably electrostatic interactions. About 10% or 15% of the phages loaded remained in the SA and SA/CS-modified gelatin beads, respectively, which was significant. Similar behavior was observed by Tabata’s group and in our related studies for the release of basic fibroblast growth factors from the gelatin hydrogel beads prepared from acidic gelatin [19,20,21]. It is important to note that one can further decrease phage release and sustain it for longer periods using more SA in the recipes, which should be optimized accordingly depending on the application of these formulations.

### 2.4. Phage Stability in Storage

Safe storage with maintaining long-term activity of phages is a very critical issue. Increasing the shelf life of phages, especially at ambient conditions, and preparing user-friendly pharmaceutical formulations for practical and much broader uses are challenging. In this study, we investigated the use of gelatin hydrogel beads as carriers/ controlled-release matrices for potential active uses of the antibacterial phages in diverse applications.

As outlined in the Introduction Section, there are several approaches to preparing, storing, and using phage-carrying formulations. Phages are biological entities that look simple but have sophisticated and highly ordered 3D structures formed off mainly proteins, which are responsible for all their activity. Due to their 3D structures, they are susceptible to loss of activity as a result of undesirable environmental stresses, including destructive lights like UV radiation and heat, changes in temperature, pH, ionic strength, and external forces [30].

Various excipients have been included in the recipes as stabilizers to protect phages, especially in long-term storage [31,32,33,34,35]. Gelatin, a natural polypeptide, has been widely studied as an excipient, usually together with other stabilizers in the recipes of phage dispersions. In those studies, gelatin was in the soluble form. In our approach presented here, we loaded phages within the gelatin hydrogel-based microbeads to develop phage-carrying/phage-releasing pharmaceutical preparations and, also, as a preserving matrix to prevent phage activity loss in both the preparation processes and long-term storage.

Here, a freeze-drying (lyophilization) process was applied to manufacture phage formulations in dry form as user-friendly and, in more convenient forms, as an alternative to liquid phage emulsion [35,36,37,38,39]. At the same time, we have been foreseeing that those formulations could be stored by keeping most of their activities for longer times at ambient temperature, which was a very challenging expectation. Lyophilization or, in other words, cryo-process is the process of cooling a material far below 0 °C, to around −30 °C in the process that we applied here, which was achieved in a liquid nitrogen medium. In this process, water ice crystals were formed and then they were sublimated by applying a vacuum leaving a porous (not wholly dried) matrix behind. After this initial low-temperature dehydration, in the second step, the mass was further dried by increasing the temperature gradually up to 25 °C. Note that these temperature changes may cause undesirable environmental stresses on phages that may lead to activity losses. The process was optimized in our pre-experiments and then applied to obtain the lyophilized gelatin-based hydrogel microbeads as described here. The average activity loss and the standard deviation of three repeated tests during this lyophilization process were obtained by the plaque assay. The average remaining activities were about 11 ± 4%, which was quite acceptable.

In this study, the storage stabilities of the following two different formulations were investigated: (i) phages in the swollen gelatin-based hydrogel beads and (ii) phages in their lyophilized forms. Both the GEL microbeads and the GEL/SA/CS beads were used. We followed the changes and the remaining phage activity such as phage titers (as PFU/mL) by plaque assay over time, up to 9 months at 25 °C.

**Figure 7:**
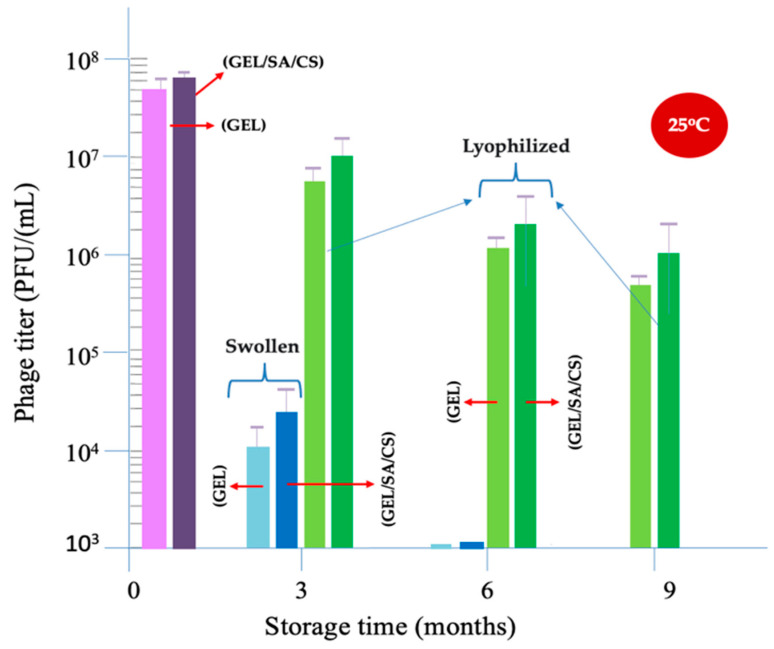
Storage stability of phages in the gelatin-based hydrogel microbeads at room temperature.

The storage test results are presented in Figure 7, which was prepared in a semi-logarithmic plotting scale. The first issue to discuss is the effect of the freeze-drying process on the phage activity losses. Note that the initial phage titer was 10^8^ PFU/mL. On the left side of the graph, two bars show the phage titers just after the lyophilization process was applied for the GEL and GEL/SA/CS microbeads. The phage activity losses are observable but not that significant. The GEL/SA/CS beads were more protective than the GEL microbeads.

There were significant activity losses of around 70% and 90% in 3 months and 6 months, respectively, in the swollen beads. There was a positive stabilization effect of using SA in the formulations, observable but not that significant. Note that there are many side functional groups (amino, carboxylic, hydroxyl, etc.) on both gelatin and alginate backbones that could be charged negatively or positively and that could react with phages electrostatically to a different extent. The isoelectric point of T4 phages is around 4.8–6.2, which represents the average charges on the protein coat at different pH [54,55,56]. However, it is known that the charges may be different at different sites on phages—like the head of T4 is negatively charged but the tails are positively charged at physiological pH. It means that phages could interact electrostatically with different side groups (like amino and carboxylic acid) on the gelatin backbone within the hydrogel beads. The interaction may not be very strong but strong enough, which makes the phages more stable during storage [54,55,56].

The effects of lyophilization on the stability of phage formulations within gelatin beads look very promising. Both the GEL and GEL/SA/CS gelatin microbeads in lyophilized forms allow for maintaining phage activity that they carry quite successfully. The activities left in these phage formulations—even after storing for 9 months at 25 °C—were acceptable, which means that they could work very effectively on the target bacteria in use. Most probably, the functional groups on both gelatin and alginate molecules interact with phages and contribute to the preservation of their activity in the dry phase. The achievements reached in this study were very promising compared with the published literature results in similar lyophilization processes, in which several coating approaches were used together with several excipients including gelatin (in soluble form) [35,36,37,38,39]. Most probably, it was because we embedded phages within gelatin-based hydrogel microbeads.

## 3. Conclusions

This article presents the results of our follow-up studies to prepare phage-controlled release formulations, hydrogel-based beads, for diverse applications with different release kinetics. Here, we have prepared both gelatin (GEL) hydrogel microbeads similar to our previous studies and the new GEL/SA/CS composite hydrogel microbeads. The basic foresight was to introduce SA within the GEL microbeads to change the cross-linking network structure within the microbeads and increase the electrostatic interactions between phages and the carrier matrices to reduce the phage release rates, due to the negatively charged alginate backbones. Using chitosan was an extra contribution, and the phage release rates were foresighted. This is actually not fully an encapsulation protocol but most probably a formation of patches made of surface alginate groups and chitosan molecules, which is an interesting concept but needs further investigation.

In this study, we have applied de-hydrothermal cross-linking because it is a very clean and eco-friendly approach achieved without using any toxic chemicals or solvents. De-hydrothermal treatment allows for inter- and/or intra-cross-linkings between these natural polymer backbones, but it also causes degradation of the backbones by forming shorter chains with extra bindings. It should be considered a niche strategy to prepare composite cross-linked networks from different natural polymers, such as polypeptide (here gelatin) and polysaccharides (here sodium alginate). The limitation is that it is almost impossible to describe the cross-linked network structure obtained after treatment.

Gelatin is a natural polymer, a denatured form of collagen, which is the main component forming the human/animal extracellular matrix. Therefore, it is highly bio-compatible and also degraded in vivo, mainly enzymatically in a very controlled manner. Cross-linking the gelatin microbeads usually decreases the degradation rate. Alginate is a polysaccharide and degraded in vivo much more slowly. However, it is expected that de-hydrothermal treatment would break down the alginate backbone and change the fate of biodegradation of the alginate parts in vivo. Comparative in vivo degradation of the GEL, GEL/SA and GEL/SA/CS hydrogel microbeads is under investigation as a follow-up of this study.

Using gelatin-based microspheres in a swollen form helped maintain phage activities at room temperature but not significantly. However, the formulations that were properly prepared from gelatin-based and lyophilized microbeads were quite successful in maintaining phage activity for about 9 months at ambient temperature with high percentages. The stability studies only demonstrate the phage activity loss in time, not the freeze-dried carrier matrices or microbeads—those were still intact even after three years of being stored in dry conditions.

## 4. Materials and Methods

### 4.1. Materials

The host and target bacteria *Escherichia coli* (*E. coli*) and its specific T4 phages were obtained from ATCC (ATCC^®^ 11303^TM^ and 11303-B4^TM^, respectively; Manassa, VA, USA). Native basic gelatin (IEP: 9.0; weight-average molecular weight: 100 kDa) was from Nitta Gelatin Co. (Osaka, Japan). Chitosan (low molecular weight) was bought from Fluka (Buchs, Switzerland). Sodium alginate and all other chemicals were purchased from Sigma-Aldrich (St. Louis, MO, USA) and were of reagent grade and used as received unless noted otherwise.

### 4.2. Propagation of the Host and Target Bacteria

*E. coli* was propagated by a classical protocol, as described previously, in Luria-Bertani (LB) medium [23,29,57,58]. Briefly, the host bacterial strain obtained from ATCC^®^ was cultured in the freshly prepared LB medium (25 g of LB in 1 L of distilled water) at 37 °C in a rotary shaker (200 rpm) until reaching the exponential growth phase. The medium was then transferred into 15 mL sterile tubes and centrifuged at 6000 rpm for about 5 min, and the pellets obtained were washed a few times and re-suspended in phosphate-buffered saline (PBS, pH 7.2). In order to describe the bacterial concentration, the suspension was diluted with PBS to obtain suspensions with different concentrations. They were then plated and cultured on LB agar (6 g agar in 400 mL of LB media) to estimate total bacterial (viable) counts (as colony-forming units, CFU) to describe the bacterial concentration in the suspension produced in CFU/mL units [58].

Phages were propagated using *E. coli* as the host prepared in the previous step by following a standard protocol [29,59,60,61,62]. Briefly, 100 μL of *E. coli* freshly prepared with a concentration of 10^8^ CFU/mL and 100 μL of T4 phage (from the stokes) with a concentration of 10^8^ PFU/mL were mixed and then incubated at room temperature for 15 min and then added to the LB medium (supported with CaCl_2_ and MgCl_2_—0.001 M each). The mixture was incubated for 6 h at 37 °C in a shaking incubator (200 rpm). For purification, the medium was first ultra-filtered through a sterile 0.22 μm filter and then centrifuged at 4 °C—13,600 *g* for 20–30 min. The purified phages were re-suspended in sterile PBS buffer (pH:7.2) or in SM buffer (0.1% gelatin, 100 mM NaCl, 8 mM MgSO_4_, 50 mM Tris-HCl, pH 7.5) and stored at 4 °C until use. Phage concentration/titers, denoted as “plaque forming unit per milliliter” (PFU/mL), were determined by a “plaque assay” technique [29,62,63,64]. Briefly, phage nanoemulsions with different concentrations were prepared by dilution of the initial phage suspension: 100 μL from each one and 400 μL of *E. coli* suspension were mixed and added to an LB medium (semi-liquid—agar 7.5 g/L) and incubated at 37 °C for 24 h, and the lysis plaques were counted.

The performances (activities) of the T4 phages propagated in the previous step—in other words, the abilities of T4 phages to destroy the target bacteria (*E. coli*)—were visualized on bacterial cultures in petri dishes (“soft agar over layers”) [59,65]. Different volumes of T4 phage nanoemulsions were dropped into the petri dishes carrying *E. coli* cultures on agar, which were then incubated at 37 °C overnight. Note that the prepared *E. coli* lawn plates were originally turbid. However, when *E. coli* were destroyed by the phages, transparent zones were formed due to the lysis of the bacteria, which was presented to exhibit the effectiveness of the phages.

The SEM micrographs of the target bacteria and its T4 phage and their interactions were also investigated using a Philips ultra plus high-resolution FESEM equipped with an in-lens secondary electron detector (Philips, Amsterdam, The Netherlands) at an operating range of 2–20 keV depending on sample charging. The suspensions/emulsions were dropped onto the silica slides, dried at room temperature, and then, images were obtained.

### 4.3. Production and Characterization of the Gelatin-Based Hydrogel Microbeads

A two-step protocol was applied to obtain gelatin hydrogel microbeads with different cross-linking degrees and cross-linked network structures by following the protocols described by Tabata and his colleagues [19,20,21,23]. In the first step, 10 mL of gelatin solution 10% *w*/*v* was heated up to 40 °C and then dropped into about 600 mL of a special olive oil dispersion phase (Wako Pure Chemical Industries, Ltd., Osaka, Japan) and then dispersed by vortex mixing to yield a water-in-oil dispersion. Gelation was achieved at 4 °C by continuous stirring of the dispersion medium, for about 1 h to reach gelation. In order to remove the residual olive oil, the gelatin gel microbeads were washed with cold acetone, also by centrifugation (at 5000 rpm at 4 °C for 5 min), and air-dried. The gelatin hydrogel beads were separated into fractions with different sizes using sieves with different size mesh, and the fraction between 32 and 53 μm was used in the following step. In the second step, the gelatin microbeads were air-dried at 4 °C and then de-hydrothermally cross-linked to obtain gelatin hydrogel beads in a vacuum incubator at 140 °C under 0.1 Torr vacuum in 24 h. These beads were denoted as the “GEL” microbeads.

For alginate modification, a very similar protocol was applied, except a gelatin/sodium alginate mixture was prepared by adding 0.1 or 0.3 g of sodium alginate into the initial 10 mL gelatin solution (with a gelatin concentration of 10% *w*/*v*), which was heated in a 40 °C water bath about 6 h by stirring to form a homogeneous gelatin/sodium alginate solution. Then, the gelatin/alginate (“GEL/SA”) gel microbeads were formed in the oil phase; the beads were washed, sieved, and cross-linked de-hydrothermally only for 24 h treatment times, as explained in the previous chapter.

The cross-linked GEL/SA hydrogel microbeads were incubated in the chitosan solution (about 1% *w*/*v*) for about 20 min to coat the surface of the beads with a chitosan layer (by polyanion/polycation complex formation between surface alginate groups and chitosan (CS) as described in the related articles [23,29,42,43,44].

The swelling ratio or water uptake of the cross-linked gelatin-based microbeads was obtained as follows: The dried hydrogel beads were swollen in distilled water at 37 °C for 24 h to reach the swelling equilibrium. Water uptake was calculated using the weights of the swollen and dried gelatin beads and presented as a percentage (% by weight). Photographs of gelatin hydrogel beads swollen in distilled water were taken with a microscope (Olympus, Tokyo, Japan). In order to calculate the average diameter of these swollen beads, the diameters of about 100 hydrogel beads were measured and the average values with standard deviations were calculated using the Image J (NIH, Bethesda, MD, USA, Version 1.51) computer program.

The Zeta potential of the GEL, GEL/SA, and GEL/SA/CS microbeads was determined using the Malvern Zeta-Sizer (Malvern instrument, Malvern, UK). For the measurement, 100 μL of microbead suspension was diluted to 4 mL with 10 mM NaCl solution, further adjusting the pH to 7.4 using 0.25 N NaOH. All measurements were taken at room temperature (25 °C) in triplicate.

The facilitated acidic hydrolysis—degradation of the gelatin-based microbeads within HCl—was studied by the following protocol [21,23]. Briefly, 5 mg of the dried cross-linked gelatin microbeads were put into a 2 mL tube containing about 750 μL of double-distilled water and allowed to fully swell for about 1 h at 37 °C. Then, 750 μL 2M HCl was added and incubated at 37 °C for different time periods to follow the degradation over time. At selected intervals, the tube was centrifuged at 5000 rpm for 5 min at 37 °C, 200 μL of the supernatant was taken, and 200 μL 2M HCl was added into the tube to continue the degradation test. Absorbance of 200 μL supernatant taken from the tube was measured at 260 nm using a UV spectrometer (Ultrospec 2000, Pharmacia Biotech, Cambridge, UK). Using the absorbance values, the total mass remaining was obtained and plotted against time to demonstrate the degradation profile.

### 4.4. Phage Loading and Release within and from the Gelatin-Based Hydrogel Beads

For the loading of phages within the gelatin-based hydrogel beads, a very simple protocol was applied. Here, we used T4 phage nanoemulsions with a concentration of 10^8^ PFU/mL. About 200 μL was added into the tube containing 2 mg of cross-linked and dried gelatin-based beads and incubated for 1 h at 37 °C. Note that all the aqueous media were completely sucked up by the dried beads because the volume of the aqueous phase was lower than the water in the wholly swollen beads—which was adjusted in the preliminary studies—meaning that loading efficiency was almost 100%. The chitosan coating was applied after the phage loading as described above.

In the phage release experiment, about 200 mg of the freshly prepared gelatin beads carrying phages were incubated in 50 mL of PBS buffer at pH 7.4 by gently shaking for up to 24 h. About 100 μL samples were withdrawn from the medium at selected time intervals (replaced with fresh medium), and the amount of active phages released was followed by a “plague assay” as described above. The cumulative amount of phages released during the incubation period was plotted against time to demonstrate the phage release kinetics.

### 4.5. Storage of Phage Formulations

To evaluate long-term storage stability, the following two different formulations containing T4 phages were studied: (i) phages either in the GEL or GEL/SA/CS (which were prepared with a GEL/SA ratio of 1.0/3.0 by weight and coated with chitosan) microbeads loaded with phages with a concentration of 10^8^ PFU/mL; and (ii) phages in the lyophilized GEL and GEL/SA/CS microbeads—lyophilization was realized after loading of phage nanoemulsions within the gelatin microbeads with a concentration of 10^8^ PFU/mL.

Lyophilization was conducted by a two-step protocol. Briefly, the gelatin-based hydrogel microbeads loaded with phage dispersion (within SM buffer) in the cryotubes were put into the liquid nitrogen medium and then transferred to the freezing system working around −30 °C. Most of the water phase first became ice crystals; then, they were sublimated by applying a vacuum leaving a porous matrix behind (not wholly dried). After this initial low-temperature dehydration, in the second step, the mass was further dried by increasing the temperature gradually up to 25 °C. The activity losses due to the lyophilization process were measured and the remaining activities are demonstrated in the bar graphs together with the standard deviation of three repeated tests.

Two different formulations described above were stored at 25 °C and their activities were assayed just after lyophilization, and after 3, 6, and 9 months. Phage titers in the microbeads were determined as described in the previous sections by a “phage plaque assay” and the phage stabilities were demonstrated as “remaining phage titers” in PFU/mL. The experiments were repeated as three independent experiments and average values with standard deviations are shown as bar graphs.

### 4.6. Statistical Analysis

The results were expressed as mean ± standard deviation (SD) from at least three independent experiments in all the tests described above. One-way analysis of variance with Tukey’s test (Origin 8.0) was used for statistical analysis. * *p* < 0.05 was statistically significant.

## Figures and Tables

**Table 1:** Properties of the GEL, GEL/SA and GEL/SA/CS hydrogel microbeads. Crosslinking by de-hydrothermal treatment for 24 h.

GEL/SA Ratio (*w*/*w*)	Water Content * (% by Weight)	D * (µm)	Zeta Potential (mV)
1.0/0.0	98.1 ± 1.4	44.5 ± 9.4	+2.5 ± 0.2
1.0/0.1	89.2 ± 1.3	38.4 ± 4.4	+2.3 ± 0.3
1.0/0.3	84.4 ± 1.1	34.4 ± 6.1	−1.8 ± 0.3
1.0/0.3 + CS			+2.9 ± 0.4

* Average ± standard deviation.

## Data Availability

The data will be available if necessary.
